# Butterflies fly using efficient propulsive clap mechanism owing to flexible wings

**DOI:** 10.1098/rsif.2020.0854

**Published:** 2021-01-20

**Authors:** L. C. Johansson, P. Henningsson

**Affiliations:** Department of Biology, Lund University, Ecology Building, Sölvegatan 35, 223 62 Lund, Sweden

**Keywords:** aerodynamics, butterflies, clap and fling, wing morphology, animal flight, unsteady aerodynamics

## Abstract

Butterflies look like no other flying animal, with unusually short, broad and large wings relative to their body size. Previous studies have suggested butterflies use several unsteady aerodynamic mechanisms to boost force production with upstroke wing clap being a prominent feature. When the wings clap together at the end of upstroke the air between the wings is pressed out, creating a jet, pushing the animal in the opposite direction. Although viewed, for the last 50 years, as a crucial mechanism in insect flight, quantitative aerodynamic measurements of the clap in freely flying animals are lacking. Using quantitative flow measurements behind freely flying butterflies during take-off and a mechanical clapper, we provide aerodynamic performance estimates for the wing clap. We show that flexible butterfly wings, forming a cupped shape during the upstroke and clap, thrust the butterfly forwards, while the downstroke is used for weight support. We further show that flexible wings dramatically increase the useful impulse (+22%) and efficiency (+28%) of the clap compared to rigid wings. Combined, our results suggest butterflies evolved a highly effective clap, which provides a mechanistic hypothesis for their unique wing morphology. Furthermore, our findings could aid the design of man-made flapping drones, boosting propulsive performance.

## 1. Background

The fluttery flight of butterflies over a sunny meadow instils fascination, yet the flight of butterflies remains somewhat a mystery. The few flight mechanistic studies performed so far on butterflies have triggered suggestions that they use a variety of unsteady aerodynamic mechanisms for their force production [1,2]. Among these mechanisms, the upstroke wing clap, first described by Weis-Fogh for insects already in the early 1970s [3], is one repeatedly reported as used by butterflies [1,4–7]. Despite the importance of this mechanism, as far as we know, quantitative measurements of the aerodynamics of the wing clap in freely flying animals are still lacking.

Apart from their characteristic fluttery flight [7,8], butterflies also perform highly directed and sustained flights as, for example, seen in migratory species [9,10], in unpalatable species [8] and during take-off [11,12]. The take-off of butterflies is typically very fast and may act as a response against potential predator threats. The fast and directed take-off flights require high force production and control and the butterflies then use wing clap to a large degree (electronic supplementary material, video S1). In this study, we have taken advantage of this behaviour and studied the aerodynamics, using tomographic particle image velocimetry, and kinematics of six individuals of silver-washed fritillaries (*Argynnis paphia*, L. 1758) during take-off flight in a wind tunnel [13] (electronic supplementary material, videos S2 and S3). The aim of this study was to determine the aerodynamics of the iconic wing clap in a freely flying animal and to determine the function and contribution of the upstroke with wing clap to the flight of butterflies.

Our findings when studying the butterflies indicated an important role of wing flexibility for the performance of wing claps. During the wing clap, the wings of the butterflies were not just two flat surfaces slamming together. At the instance of the clap, when the two wings meet, the wings had a reversed camber, likely owing to their flexibility. This resulted in a ‘cupped' clap where the leading edge of the wings intercepted before the central parts of the wings, while the rear wing-bases were kept close together at the body (see electronic supplementary material, videos S1 and S2). This peculiar shape of the wings during upstroke has been noted previously for butterflies [6], but the mechanistic function of it has not been further explored. We hypothesized that a cupped clap with flexible wings, essentially forming an air pocket in the late stages of the clap, results in a larger impulse because the wings will be affecting a bigger air volume at the final critical instance of the clap. In addition, we predicted the cupped clap of the flexible butterfly wings to be more efficient than a rigid wing clap since the cupped wings contain the air volume better when the wing edges form a more closed structure. The cupped shape could also reduce detrimental ‘air leakage' between the leading edges of the wings, which would otherwise result in a reaction force on the animal in an undesired direction (e.g. downwards or backwards). To test these hypotheses, we constructed a mechanical clapper with two sets of wings, one rigid and one flexible.

## 2. Methods

### 2.1. Study animals

We used six individuals of silver-washed fritillaries (*Argynnis paphia*), which were wild caught in the meadows around the departmental field station, Stensoffa, in southern Sweden (55°41'42.5″ N 13°26'51.2″ E). We weighed the butterflies before and after the experiments and performed measurements of wing morphology from photographs of the butterflies on reference grids after the experiments (electronic supplementary material, table S1). Between flights butterflies were kept in large net cages and were fed honey water solution.

### 2.2. Experimental procedure

We measured kinematics and aerodynamics of the butterflies in the Lund University wind tunnel [13] as they took off voluntarily from a feeder in the centre of the tunnel test section (electronic supplementary material, video S2). The feeder was a circular platform (diameter of 10 mm) aligned horizontally with the freestream flow to minimize flow disturbance. The tunnel was running at approximately 2 m s^−1^ to keep the animals at a reasonable distance from the light sheet and to allow for a decent propagation speed of the wake to reach the measurement plane, 180 mm downstream of the platform. Running the tunnel at a low speed also kept the seeding particles (DEHS droplets, approx. 1 µm diameter) suspended and evenly distributed in the air.

### 2.3. Particle image velocimetry

We used a tomographic particle image velocimetry (tomoPIV) set-up with four high-speed cameras (LaVision Imager pro HS 4M, 2016 × 2016 pixels) aiming obliquely from above and behind at a transverse (*yz* plane) light sheet (approx. 5 mm thick) produced by a laser (LDY304PIV laser, Litron Lasers Ltd, Rugby, UK) (electronic supplementary material, figure S1A). In tomoPIV, 3D velocity vectors are determined in a volume based on the movement of particles for which positions are determined from images of multiple cameras. Images of the seeding particles were captured at a frame rate of *f_L_* = 640 Hz. To allow for the 3D triangulation of the particles' position the system was calibrated using the Lavision Type 22 calibration plate, followed by the tomographic self-calibration routine in Davis. To be able to capture the rapid take-off of the butterflies, we had to anticipate the behaviour of the butterfly since we could only work with a pre-triggered set-up. Due to a ramp-up time of the laser and the short distance between the feeder and the laser sheet success rate was relatively low. Cameras were calibrated and PIV images were analysed in Davis 8.3.1 (LaVision Gmbh, Göttingen, Germany). In total, we analysed 25 sequences containing 1–3 wingbeats each. Not all wingbeats were possible to analyse in all aspects (e.g. in some we could not isolate the upstroke wake from the rest of the wake). For the analysis, we defined a right-handed orthogonal coordinate system with the *x*-axis aligned with the freestream direction, the *y*-axis in the spanwise direction and the *z*-axis in the vertical upwards direction.

TomoPIV raw images from the four cameras were pre-processed using subtract sliding minimum over 5 pixels, intensity normalization to local average of 300 pixels, Gaussian 3 × 3 smoothing, and multiplication with a factor 10. The pre-processed images were then used to calculate a 3D particle space using the FastMART routine. We then used the 3D direct correlation routine with decreasing box size starting at 64 × 64 × 64 with 50% overlap (4 × 4 × 4 binning), followed by 48 × 48 × 48 with 50% overlap (2 × 2 × 2 binning), followed by 32 × 32 × 32 boxes with 50% overlap (no binning) and for the final step 22 × 22 × 22 boxes with 50% overlap (three passes, no binning). We used a two-times remove and insert filter (5 × 5 × 5 voxels) to remove erroneous vectors, followed by a three-times smoothing (3 × 3 × 3 voxels) between rounds (not smoothing the final vector fields). The resulting vector fields were post-processed using a remove and insert filter (3 × 3 × 3 voxels). Empty spaces were filled by interpolation and the final vector fields were smoothed with a 3 × 3 × 3 Gaussian filter. The final vector volume size was approximately 4.4 × 320 × 250 mm and approximately 4 × 220 × 170 vectors, resulting in a vector spacing of approximately 1.45 mm for all three axes (6.8 vectors cm^−1^). For all further analyses, only the second plane (in the *x*-dimension) in the volume was used.

### 2.4. Butterfly analysis: pre-processing

The butterflies were typically performing a turn as they were taking off. Because of this, during the course of the turn, the wake from the animal becomes increasingly angled towards the transverse (in relation to the wind tunnel) measurement plane. To allow for a proper analysis where both the thrust along the flight direction and the vertical forces could be determined we decided to ‘straighten' the wake of the butterfly i.e. to align our analysis plane perpendicular to the flight path. To do this, we first generated 3D matrices of the vector fields with a spacing between vectors in the measurement plane of d*y*×d*z*, and d*x* = *U*/*f_L_* in the out of plane direction, were *U* was the freestream flow determined from calculating the mean flow of undisturbed areas of the vector fields. We then visualized the wake using iso-surfaces of total vorticity and while viewing the horizontally projected 3D reconstructed wake from above, we manually clicked three points at the wake centre along the flight path (beginning, middle and end). These three points were then used to fit a circle representing an approximation of the turning flight path (electronic supplementary material, figure S1C). The 3D wake volume was then sliced in equi-angular spaced planes along the circular path with the centre determined by the fitted circle. The velocity and vorticity were interpolated onto the planes using the *interp3* function in Matlab. We made the resulting vector fields the same resolution in the *y'z* plane as the original *yz* planes and each sequence the same number of frames as the original dataset. The distance between the new planes in the new wake *x*’ direction was therefore determined by the tightness of the turn and was taken into account in all following calculations. From an analysis perspective, this procedure corresponds to straightening out the flight path, and the procedure is illustrated in electronic supplementary material, figure S1D. To make sure our force estimates remained correct, we calculated the local freestream *U*_∞_’, the speed perpendicular to the *y*’*z*-plane, along the *y'*-axis and used it in the force calculations (see below). In the end, the only function of this procedure is to make sure that we measure forces and impulses relative to the flight path rather than the wind-tunnel based coordinate system.

### 2.5. Butterfly analysis: force and impulse

To evaluate flight performance, we estimated forces and impulses from the straightened wakes. We estimated the net thrust/drag (*T*_net_) in each frame using a wake deficit model,

2.1 $$T_{\mathrm{net}}=\rho \int \int_{\begin{array}{c} \mathrm{wake}\\ \mathrm{area} \end{array}}u^{\prime }(y^{\prime },z)\cdot (U_{\infty }^{\prime }(y^{\prime },z)+u^{\prime }(y^{\prime },z))\;\text{d}y^{\prime }\text{d}z,$$

where *ρ* is the air density (1.2 kg m^−3^), the free-stream velocity ($U_{\infty }^{\prime }$) varies along *y′* and *u′* is the out of plane induced velocity component (i.e. measured velocity - $U_{\infty }^{\prime }$). These, and the following, calculations thus take into account the variable out of plane velocity caused by the turning flight of the butterfly. For these measurements, we used a manual masking, enclosing the wake with a polygon (electronic supplementary material, figure S1B). The size and shape of the polygon was determined by examining the out of plane velocity, attempting to include all the flow visibly affected by the animal, but as little as possible of the background noise. The backward motion of the wings relative to the body during the upstroke as well as the self-induced velocity of the upstroke wake results in an overlap of the downstroke and upstroke wakes in the flight direction (*x*′), i.e. in a single plane we may have wake structures belonging to two separate downstrokes and an upstroke. This means that it is impossible to separate the effect of the downstroke and upstroke regarding thrust/drag in our wake deficit measurements.

For the impulse measurements, we used an automatic 3D masking routine in combination with the manual masking (electronic supplementary material, figure S1B) described above for the net thrust measurements to determine the wake area and to avoid influencing the measurements with background noise. The automatic 3D masking used a threshold value of 3D vorticity (set by the operator) (Matlab, imbinarize), followed by a removal of small disconnected 3D patches (determined by operator based on background noise level) (Matlab, bwareaopen) and a hole filling routine (Matlab, imclose with a sphere size 7). To smooth the mask and make sure, we did not miss any vorticity we then conducted an erosion/dilation procedure (Matlab, imerode and imdilate) using cuboids of 2 × 2 × 2 and 7 × 7 × 7, respectively. The mask contained all wake structures, visible to eye and the procedure reduced the variation in the data very effectively while at the same time capturing most (if not all) of the vorticity generated by the animal.

The butterflies clapped their wings together or close to each other at the end of the upstroke. Since the upstroke wake is vertically oriented, the time taken to generate it is not possible to determine from our wake data (i.e. the wake structure that have taken half a wingbeat to generate ends up in a few frames). Therefore, force (i.e. rate of change of impulse) could not be estimated. To circumvent this problem and allow for determining the impact of the clap, we instead estimated the impulse of the vortex ring generated during the upstroke phase of the wingbeat determined from the top view of the 3D volume (*x*′*y′z*). In these measurements, we used the manual masking to isolate the upstroke wake from other wake structures. The impulse aligned with the flight direction (*I*_UST_) was calculated as

2.2 $$I_{\mathrm{UST}}=\rho \Delta z\overset{N_{z}}{\underset{n_{z}=1}{\sum }}\int \int_{\begin{array}{c} \mathrm{wake}\\ \mathrm{area} \end{array}}{\omega^{\prime }}_{z}(x^{\prime },y^{\prime },n_{z})\cdot s_{y^{\prime }}(x^{\prime },y^{\prime },n_{z})\cdot \text{d}x^{\prime }\text{d}y^{\prime },$$

where vorticity $\omega_{z}^{\prime }$ is in the local plane (*x*′*y*′), $s_{y^{\prime }}$ is the distance to the centre of the wake in the *y*′-direction, Δ*z* is the vertical distance between the measurements based on vector resolution and *n*_z_ is the horizontal plane number. Note that the area used in the calculations varies along y′ due to the curved path of the original trajectory. Impulse directed to the sides (*I*_USS_) was estimated as

2.3 $$I_{\mathrm{USS}}=\rho \Delta z\overset{N_{z}}{\underset{n_{z}=1}{\sum }}\int \int_{\begin{array}{c} \mathrm{wake}\\ \mathrm{area} \end{array}}{\omega^{\prime }}_{z}(x^{\prime },y^{\prime },n_{z})\cdot s_{x^{\prime }}(x^{\prime },y^{\prime },n_{z})\cdot \text{d}x^{\prime }\text{d}y^{\prime },$$

where $s_{x^{\prime }}$ is the distance to the centre of the wake along the *x*′-direction, back-calculated to the original coordinate system. As in equation (2.2), the area used varies along *y′*. We then calculated the total upstroke clap impulse *I*_US_ as the vector sum of *I*_UST_ and *I*_USS_.

Vertical impulse of the upstroke was calculated as

2.4 $$I_{\mathrm{USV}}=\rho \Delta z\overset{N_{z}}{\underset{n_{z}=1}{\sum }}\int \int_{\begin{array}{c} \mathrm{wake}\\ \mathrm{area} \end{array}}^{\;}\omega_{x}^{\prime }(x^{\prime },y^{\prime },n_{z})\cdot s_{y^{\prime }}(x^{\prime },y^{\prime },n_{z})\cdot dx^{\prime }dy^{\prime },$$

where $\omega_{x}^{\prime }$ is in the local plane (*x*′*y*′).

Due to the spatial overlap of the wake of the downstroke and upstroke along the *x*' direction we could not directly estimate the contribution of the upstroke and downstroke to the vertical and thrust impulse. Instead, we estimated the vertical (*I*_WBV_) and thrust impulse (*I*_WBT_) of the entire wingbeat as

2.5 $$I_{\mathrm{WBTz}}=\rho \Delta x^{\prime }\overset{N_{x}}{\underset{n_{x}=1}{\sum }}\int \int_{\begin{array}{c} \mathrm{wake}\\ \mathrm{area} \end{array}}{\omega^{\prime }}_{z}(y^{\prime },z,n_{z})\cdot s_{y^{\prime }}(y^{\prime },z,n_{x})\cdot \text{d}y^{\prime }\text{d}z\;z,$$

2.6 $$I_{\mathrm{WBTy}}=\rho \Delta x^{\prime }\overset{N_{x}}{\underset{n_{x}=1}{\sum }}\int \int_{\begin{array}{c} \mathrm{wake}\\ \mathrm{area} \end{array}}{\omega^{\prime }}_{y}(y^{\prime },z,n_{z})\cdot s_{z}(y^{\prime },z,n_{x})\cdot \text{d}y^{\prime }\text{d}z\;$$

2.7 $$\text{and}\;I_{\mathrm{WBT}}=\frac{I_{\mathrm{WBTz}}+I_{\mathrm{WBTy}}}{2},$$

where *I*_WBTz_ and *I*_WBTy_ are the *I*_WBT_ calculated based on respective vorticity components, *s*_z_ is the is the distance to the centre of the wake in the *z*-direction and Δ*x′* is the local distance between the planes in the straightened wake

2.8 $$I_{\mathrm{WBV}}=\rho \Delta x^{\prime }\overset{N_{x}}{\underset{n_{x}=1}{\sum }}\int_{\begin{array}{c} \mathrm{wake}\\ \mathrm{area} \end{array}}{\omega^{\prime }}_{x}(y^{\prime },z,n_{z})\cdot s_{y^{\prime }}(y^{\prime },z,n_{x})\cdot \text{d}y^{\prime }\text{d}z.$$

We then subtracted the impulse of the upstroke (*I*_UST_ and *I*_USV_) from the impulse of the entire wingbeat to get the impulse generated by the downstroke.

### 2.6. Butterfly kinematics

In addition to the PIV measurements, we conducted a kinematic analysis using a stereo set-up with two high-speed cameras (High-SpeedStar3: 1024 × 1024 pixels, running at 640 Hz), looking down on the animal obliquely from above/in front and straight from above. We used clicking_gui_two_cams, a custom written Matlab software by Dr Simon Walker [14], to digitize/track a point on the head, left and right forewing tips (apex) and left and right tornus in the two views (electronic supplementary material, figure S2A). The camera views were calibrated using the calib–gui routine accompanying the digitization software and the coordinates of the two views transformed into 3D coordinates in real space (electronic supplementary material, figure S2B). Using the head marker, we estimated the mean flight speed along the curved path.

We determined the transition between upstrokes and downstrokes from the angle between the two forewings in the stroke plane. Stroke plane was defined using a PCA-analysis (Matlab) of the position of the left- and right-forewing tips relative to the head position. The first two principle components captured variation in the data due to the distance between the wing tips and the direction of the wingbeat and hence principle component 3, which by definition is perpendicular to the other principle components (i.e. describes the normal to the plane the wings sweep through) was used as the stroke plane. We verified that the orientation of the plane we arrived at was reasonable by plotting it along with all the wing tip coordinates of the sequence. We hypothesized that the volume of air trapped between the wings and the speed at which this air was pressed out by the wings as they clap together, along with how close the wings were brought together at the end of upstroke, would be the main drivers affecting the strength of the impulse. To test this hypothesis, we considered tip-to-tip amplitude of the wings to be the main factor determining the volume of air affected by the wings and wing angular velocity to be the main factor determining the induced velocity of the air produced during the upstroke and clap phase. We considered the angle between the wings to account for the closeness of the wings at the end of the upstroke. The start and end of the upstroke was used to determine the angular amplitude (*θ*) in the stroke plane (i.e. projected onto the stroke plane). We calculated the mean angular velocity of the wings relative to each other ($\dot{\varphi }$) during the upstrokes by derivation of a spline function fitted to the data of the angle between the wings (left wing tip – head – right wing tip) over time.

### 2.7. Clapper: design and experimental procedure

To test the hypothesis that wing flexibility would improve performance through generating a cupped wing shape, we constructed a mechanical ‘clapper' inspired by the butterfly cupped wing clap. In order to make it possible to generalize the results and test if flexibility alone has an effect, our ambition was to construct a mechanical model that was the simplest rendition of the wing-clap system and not to exactly mimic properties of real butterfly wings (such as specific kinematics, wing flexibility and morphology e.g. two interacting wing pairs versus a single wing pair). The clapper wings were constructed in two sets of wing pairs in the shape of symmetrical right triangles, one rigid and one flexible (electronic supplementary material, figure S3C). The flexible wings were supported along the catheters of the triangle while the hypothenuse was free to deform. The wings performed a rotary motion around one of the catheters of the triangle, around a shaft made from an M3 threaded metal rod which was running through the centre of a metal hinge, until they clapped together i.e. it had a single degree of motion. On the two hinge plates, the wing materials were attached with epoxy glue. The two wing sets were made using either a latex membrane (0.25 mm thick) or balsa wood (1 mm thick). The membrane wings were supported along the two right angled sides (along the central shaft and the leading edge) with 3 mm carbon fibre tubes along the leading edge (electronic supplementary material, figure S3C). In order to keep the thickness of the leading (upper) edge of the two wing types the same, the solid wings were fitted with a 2 mm carbon fibre tube along the leading edge, which together with the 1 mm thickness of the balsa wood gave 3 mm thickness in total, same as that of the membrane wing (50 × 50 mm right triangle). This is the simplest form, both in terms of wing shape and wing kinematics, resembling and functioning in a manner similar to the wing clap in butterflies. We believe that the triangular shape, contrasting the square wings previously used to study wing clapping [15,16], is a better approximation of the butterfly wings as illustrated in electronic supplementary material, figure S3A (note that this illustration only aims to show the triangular shape in relation to the butterfly wing shape and not to reflect the true scales of the clapper and butterfly).

The wings were actuated with a servo (Hitech HS-82MG) fed with 6 V from a power supply. The servo was fitted with a two-sided servo arm allowing the servo to push one metal rod and simultaneously pull another. The two rods were connected to screw eyes on two 3 mm carbon fibre tubes attached to and extending in front of the clapper wings so that when the servo was turning it would close the gap between the two wings by pushing one wing and pulling the other (electronic supplementary material, figure S3C). The leading edges of the wings are thus moved only due to the rotation around the central shaft and due to the low freestream speed (see below) the angle of attack is determined only by the rotational speed and the deformation of the wing. The servo was controlled with an Arduino Uno using the ‘VarSpeedServo' library (downloaded at www.arduino.cc) and the code was compiled using Arduino 1.8.5. The clapping motion of the two sets of wings was measured by digitizing the tips of the wings throughout the motion in two stereo-calibrated views of the PIV-cameras that showed the clapper in the background. For both wing sets, the two wings were clapped together at a rotational speed of approximately 1000° s^−1^ and the Arduino control ensured high consistency of the clapper performance (electronic supplementary material, figure S3B).

### 2.8. Clapper analysis: impulse

We measured the induced air flow created by the wings during clapping using the same tomoPIV set-up as for the butterflies. The clapper experiments were performed at near still air, but to keep seeding particles suspended and evenly distributed, the tunnel was running at a low speed (approx. 0.1–0.2 m s^−1^). Consequently, the variation in the background flow was high relative to the mean and often displayed weak gradients in velocities over the measurement area. Therefore, we decided to improve the procedure of background flow subtraction beyond the standard simple subtraction of the mean flow which is adequate for higher flow speeds but may be too crude for slow speeds. We removed the background flow by subtracting the velocity estimated from a plane fitted to each of the velocity components over the entire frame (Matlab, fit, poly11). Before fitting the plane, we applied a mask generated using an automated threshold filter on the 3D vorticity in each of the measurement volumes, such that the wake of the clapper was excluded when fitting the plane to the background flow. The coefficients of the fitted planes were smoothed over the sequence, to reduce the effect of random errors. This way we captured any systematic variation in the background flow over the sequence. We estimated the impulse aligned with the wind tunnel flow direction in each frame as

2.9 $$I_{\mathrm{clapT}}=\rho \int \int \int_{\begin{array}{c} \mathrm{wake}\\ \mathrm{volume} \end{array}}\omega_{z}(y,z)\cdot s(y,z)\;\text{d}x\;\text{d}y\;\text{d}z,$$

where *ω_z_* is vorticity along the *z*-axis, *s* is the distance to the centre of the wake along *y* and in the vertical direction as

2.10 $$I_{\mathrm{clapL}}=\rho \int \int \int_{\begin{array}{c} \mathrm{wake}\\ \mathrm{volume} \end{array}}\omega_{x}(y,z)\cdot s(y,z)\;\text{d}x\;\text{d}y\;\text{d}z,$$

where *ω_x_* is the vorticity in the local plane (*yz*). The convection time of the wake through the measurement plane is determined by a combination of background velocity and the self-induced velocity of the vortex ring. Since we are unable to determine the latter in our set-up with a very thin tomo volume (it would require a 3D tracking of the vortex structures over time), we cannot determine the true size of *U*/*f* normally used to define the volume of air having a specific velocity or vorticity. Therefore, it is not possible to determine the total impulse of the clap. Instead, we determined the mean impulse over the number of frames that contained the majority of the wake impulse (using the voxel size in the tomo volume, d*x*d*y*d*z*, for the individual impulse calculations). The start and end of the sequence (i.e. the frames used for the mean impulse calculations) were determined by eye, but given the narrow confidence interval of the average sequences we consider this procedure robust.

### 2.9. Clapper analysis: kinetic energy

In addition to estimating the impulse, we calculated the kinetic energy in each of the frames as

2.11 $$E=\frac{1}{2}\rho \;\int \int \int_{\begin{array}{c} \mathrm{Wake}\\ \mathrm{volume} \end{array}}(v_{x}^{2}+v_{y}^{2}+v_{z}^{2})\cdot \left(1+\frac{v_{x}}{\text{d}x\cdot f}\right)\;\text{d}x\;\text{d}y\;\text{d}z,$$

where *v*_x_, *v*_y_ and *v*_z_ are the induced velocity components in *x,y,z* directions and *f* is the frame rate.

The volume of the voxels that the measurements are made on is determined firstly by the spacing between the vectors but is then adjusted to account for the induced velocities. Since the freestream flow was small, the induced velocities were relatively high in relation and therefore affected the volume of air that should be considered. This is accounted for in the rightmost term in equation (2.11) where an induced velocity higher than *d*_x_*f* adds to the volume and a lower one decreases it.

For the kinetic energy calculations, we first analysed the full flow field (wake and background) and then subtracted the kinetic energy associated with variation in the background flow. To estimate the kinetic energy in the background flow, we determined the standard deviation around the mean background velocity for 15 frames before the wake had arrived at the measurement plane and 15 frames after the wake had passed, and used a linear model to interpolate and estimate the standard deviation in each frame. We had no reason to suspect that the background noise would change over the course of a measurement, but this way we could carefully determine the noise level with minimum risk of accidentally basing it on a temporary fluctuation. For each frame, we estimated the mean energy in the background using 1000 Monte Carlo simulations of all vectors in each frame based on the noise level estimated from the standard deviation in the background flow.

### 2.10. Statistical analysis: butterfly data

The test for correlation between the mean impulse along the flight direction (thrust impulse) created during the upstroke and the total net thrust of the complete wingbeat was performed using a linear mixed-effects model (lmer, in the lme4 package, v. 1.1–21) in R (R Core Team (2017). Vienna, Austria). We set the net thrust per sequence as response variable and the upstroke impulse as predictor. We added individual as a random variable and used number of wingbeats in a sequence as weight. For this analysis, we used 23 sequences, in which we could isolate the upstroke wakes.

The test for correlation between closeness of the wings at end of upstroke along with angular velocity during the upstroke multiplied by the upstroke angular amplitude and the upstroke impulse was performed using a mixed model in R with upstroke impulse as response variable and angular velocity times amplitude as predictor. Since the butterflies used the clap to a larger degree in the initial wingbeats, we added wingbeat number (1, 2 or 3) as a random variable in the model. We also added sequence nested under individual as a random factor in order to control for the potential effect using multiple wingbeats from the same sequence. For this analysis, we used 28 wingbeats, where we had complete data for both kinematics and upstroke impulse.

Ninety-five per cent confidence intervals (CI) were calculated using the R-function confint (in the lme4 package, v. 1.1–21) with method set to ‘Wald' and *R*^2^-values were calculated using r.squaredGLMM from the MUMln package (v. 1.43.15).

### 2.11. Statistical analysis: clapper data

The average curves with CIs were estimated by interpolating values for each of the sequences to a normalized timescale, determined by the start and end frames of the wake impulse and the mean number of frames of all the wakes. For the impulse curves, we used the impulse aligned with the direction of the mean impulse in the *xz*-plane, i.e. that can be used for thrust or weight support. For each normalized time, we estimated the mean impulse for all the sequences and the confidence interval as 2 × SEM as well as the mean wake energy and corresponding confidence interval.

We tested for differences between the two wing types regarding average impulse in the *xz*-plane, average kinetic energy per unit impulse and angle of the mean impulse relative to the horizon using a mixed model in R (R Core Team (2017). Vienna, Austria). We set wing type as fixed factor and controlled for the weak background flow by including it as a random factor.

## 3. Results

### 3.1. Butterfly take-off

**Figure 1.**
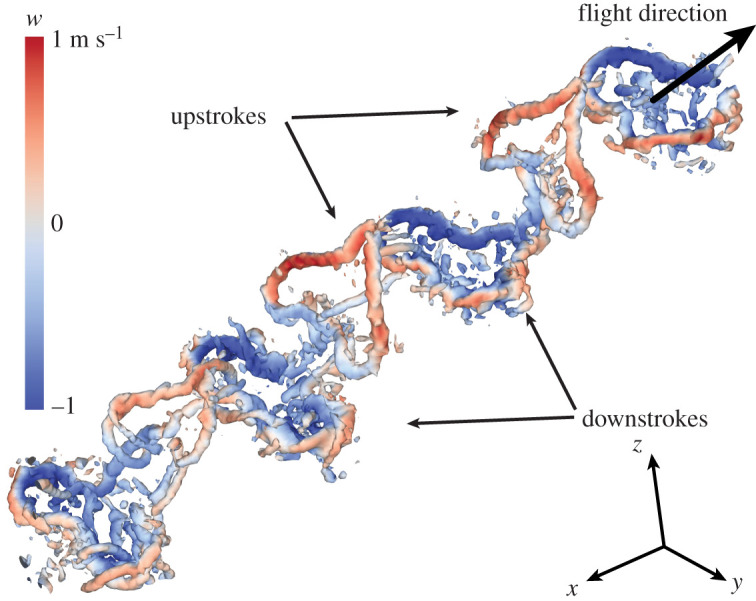
Downstroke is used for weight support while upstroke is used to generate thrust. Upstroke and downstroke wakes are clearly separated during take-off flight in a butterfly. The wake vortices of the downstrokes, encircling an area of downwash (blue), are oriented in a way indicating generation of mainly vertical force and some side force (i.e. horizontal rings with some sideways tilt). The upstroke wake vortices are instead vertically oriented, indicating mainly thrust production. Wake vortices are illustrated as iso-surfaces of *Q* (=4000), a measure of rate of rotation relative to rate of shear in the flow, coloured by downwash (*w*, induced flow in the *z*-direction). The wake is seen obliquely from above and behind, with the butterfly flying into the image and to the right. See electronic supplementary material, figure S4 for a rotatable and zoomable version of the figure.

**Figure 2.**
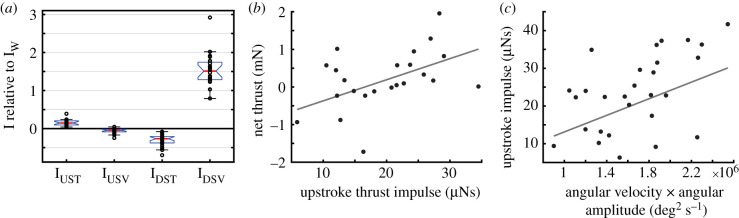
Upstroke propels the butterfly. (*a*) The contribution of the upstroke and downstroke to the thrust and weight support differs, as seen from wake impulse measurements relative to the vertical impulse required to maintain weight support (IW). The upstroke thrust impulse (I_UST_) show positive values (i.e. propelling the butterfly forwards), while the downstroke thrust impulse (I_DST_) is negative. The downstroke vertical impulse (I_DSV_), on the other hand, shows values above one indicating climbing and upwards accelerating flight, while the upstroke vertical impulse (I_USV_) shows small negative values indicative of a negligible negative contribution to weight support. The red line marks the median value, the top and bottom of the box marks the 75 and 25% range of the data and the whiskers define the 99.3% data range assuming normal distribution. (*b*) The more upstroke thrust impulse generated during an upstroke (*N* = 23) the higher the net thrust measured in the wake, indicating that the upstroke is important for the thrust production in our butterflies. The sequence average net thrust measured in the wake is estimated using a wake deficit model, while the average upstroke thrust impulse is measured using a vorticity integration (see Methods). (*c*) The amount of upstroke impulse, vector sum of thrust and sidewise directed, generated during individual upstrokes (*N* = 28) depend on the kinematics of the wingbeat and shows a positive correlation with the product of angular amplitude and mean angular velocity during the upstroke. These kinematic parameters correlate with the volume of air affected by the wings and the speed the air is accelerated to. The lines in *b* and *c* are the regressions generated using the mixed linear model (see Statistics in Methods) and the equations are found in the results.

During take-off, we found that the butterflies primarily used the downstroke for generating weight support and the upstroke for generating thrust (figures 1 and 2*a*). At the beginning of the downstroke the wings are peeled apart, generating a unified start vortex (or connected wing tip vortices from the left and right wings) resulting in a single vortex ring, generated jointly by the four wings during each downstroke (figure 1; electronic supplementary material, figures S4 and S5). The general direction of the force vector during this phase is mainly upwards and slightly sideways, as indicated by the horizontally and slightly sideways tilted vortex ring. The sideways tilt comes from that the butterflies were typically turning as they were taking off, which meant there was also a lateral force component corresponding to the radius of the turn.

**Figure 3.**
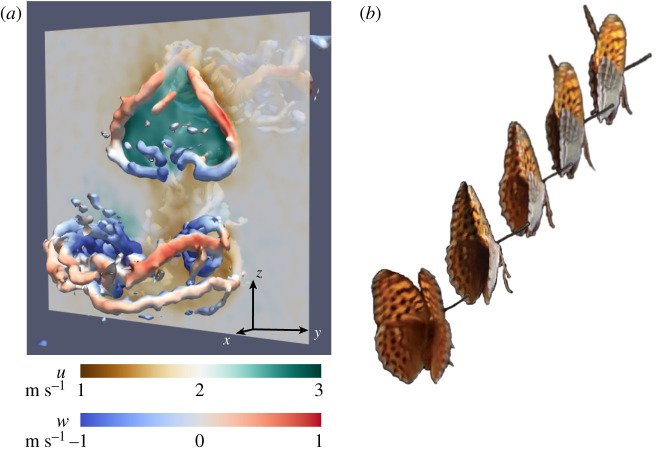
Upstroke generates thrust. (*a*) A transect through the upstroke wake shows an accelerated induced flow (green) in the freestream direction (*u* in the *x*-direction) illustrating the thrust generated during the upstroke and clap of a butterfly flying at approximately 2 m s^−1^. Wake vortices are illustrated iso-surfaces of *Q* (=4000), coloured by downwash velocity (*w*, in the *z*-direction). (*b*) A sequence of images of a freely flying butterfly in the late stages of the upstroke, illustrating the cupped clap caused by the flexible wings when the leading edges of the fore wings meet before the trailing edges.

The initial phase of the upstroke is quite weak, while the end of the upstroke when the wing clap occurs generates a vertically and spanwise aligned vortex structure. The force vector of the upstroke is directed forwards, reflecting mainly thrust production (figures 1 and 2*a*; electronic supplementary material, figure S4). The butterflies typically performed the most pronounced wing clap during the initial wingbeats of each take-off flight. When the two wings clap together, induced velocities in the wake are high in the rearward direction (and limited in the lateral directions) reflecting high thrust production (figure 3).

We measured the impulse of the vortex ring generated during the upstroke and clap phase (figure 3) and found that the impulse along the flight direction (upstroke thrust impulse, *I*_UST_) is significantly correlated with the net thrust (*T*_net_, calculated from the flow deviation from free stream speed) of the complete wingbeat (mixed model, individual as random variable and sequences weighed by number of wingbeats: *T*_net_ = 56.13 (CI ± 37.75) · *I*_UST_ − 9.3 × 10^−4^ (CI ± 9.04 × 10^−4^), *p* = 0.0036, $R_{\mathrm{model}}^{2}=0.534$, $R_{I_{\mathrm{UST}}}^{2}=0.217$, *N* = 23, see). Values are given with 95% CI. This suggests that the upstroke and clap phase is contributing significantly to overall thrust (figure 2*b*). The difference between the two *R*^2^ values, $R_{\mathrm{model}\;}^{2}$(full model including individual as random variable) and $R_{I_{\mathrm{UST}}}^{2}$ (impulse variable) and that some of the variation is explained by differences between individuals.

The upstroke and clap phase always produced a clear net thrust impulse (24 out of 24 sequences, figure 2*a*), while the other parts of the wing stroke on average produced a net negative thrust impulse (22 out of 24 sequences, figure 2*a*), which is a corroboration of the importance of the upstroke and clap for thrust production. In the data from our study, the upstroke very rarely contributed to weight support (only in 1 out of 24 sequences, figure 2*a*). Weight support was instead mainly contributed by the downstroke (figure 2*a*), demonstrating a clear separation in function between the downstroke and upstroke. The upstroke thrust impulse was on average approximately 11% of the downstroke vertical impulse, indicating a lift to thrust ratio of 9.4 for this non-steady flight behaviour.

We found a variation in the useful, horizontally directed, impulse (*I*_US_, thrust and side impulse) created during the upstroke and clap phase between different wingbeats and flight sequences. When we tested the model including both the angle between the wings at end of upstroke and the product term between amplitude and angular velocity, the closeness angle did not have a significant explanatory value. We therefore excluded it from the analysis and tested the reduced model with only the product term between amplitude and angular velocity. We found a positive correlation between upstroke impulse (*I*_US_) and angular tip-to-tip amplitude (*θ*) multiplied by angular velocity ($\dot{\varphi }$) of the wings during the upstroke (mixed model, wingbeat number, individual and sequence nested within individual as random variables:, *I*_US_ = 1.10 × 10^−11^ (CI ± 0.52 × 10^−11^) · (*θ* · $\dot{\varphi }$) + 2.12 × 10^−6^ (CI ± 12.8 × 10^−6^), *p* = 0.000033, *R*^2^_model_ = 0.766, $R_{\theta \dot{\varphi }}^{2}=0.180$, *N* = 28), as expected, showing that the effect of the wing upstroke and clap phase has a rather straight-forward mechanistic basis (figure 2*c*), but that there is variation between individuals and potentially between wingbeats in the sequence in how they perform the upstroke and clap that explain how much impulse is generated.

### 3.2. Clap aerodynamics

**Figure 4.**
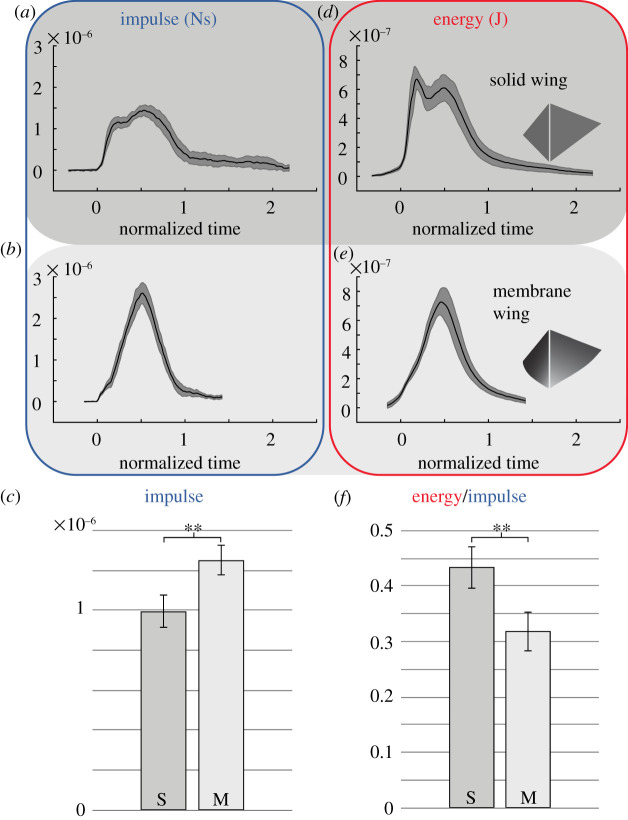
Flexible wing improves force and efficiency of wing clap. (*a*) How the impulse varies over a normalized clap wake differs between a solid wing and (*b*) a flexible membranous wing with indications of a double peak in the solid wing curve. (*c*) Comparing the impulse of the two wings we find a higher mean impulse generated by the membranous wing. (*d*,*e*) The associated kinetic energy in the wake show a similar pattern as the impulse. (*f*) The mean kinetic energy divided by the mean impulse is lower for the membranous wing demonstrating that the flexible wing is more efficient at generating the impulse than the solid wing. The *x*-axis of panels (*a*,*b*,*d*,*e*) represents normalized time, where 0 is the time of first impulse in the wake and 1 is the time the distinct clap wake has passed through the measurement plane. The dark-grey area around the curves in (*a*,*b*,*d*,*e*) shows the variation in the data as 2× SEM. Measurements of individual claps can be seen in electronic supplementary material, figure S3.

We found that the flexible wings performed significantly better than the rigid. The flexible wings generated a 22% higher average impulse than the rigid wings (mixed model, *p* = 0.0084) (figure 4*c*) and showed a dramatically improved performance in terms of efficiency, 28% lower average wake energy per unit impulse (mixed model, *p* = 0.0026) (figure 4*f*). The direction of the impulse in relation to the leading edge of the wings at the end of the clap did not differ between the two wings (mixed model, *p* = 0.26). The solid wing showed a double peak in impulse (figure 4*a*; electronic supplementary material, figure S3D) and energy (figure 4*d*; electronic supplementary material, figure S3E) which was not seen for the flexible wing (figure 4*b*, *e*; electronic supplementary material, figure S3F, G), indicating that the wake structure of the two wings differ (possibly due to the formation of double vortex rings, which was seen in the wake of the solid wing, but not obvious in the wake of the flexible wing). The wake of the clap in the freely flying butterfly did not show double ring structures, which suggest the possibility that flexible wings affect the vortex formation and since a single vortex structure results in a more uniform flow than two sub-structures, there is a direct effect on the efficiency of the clap as well.

## 4. Discussion

The flexible membrane wings in our study showed a substantial increase in both mean impulse (+22%) and efficiency (+28%) compared with the solid wings during the clap. This demonstrates that apart from wing shape and kinematics, the properties of the wing material in itself can, by resulting in a cupped wing shape during the clap, have substantial impact on performance of clapping wings (figure 4). Wing flexibility has proven to improve aerodynamic performance of insect wings also during other phases of the wingbeat (e.g. [15]) and the evolutionary step to use flexible wings, evolved for increased lift production efficiency, to generate a cupped wing shape during the clap may not be very far. The wings in our clapper study by design operate at angles of attack (angle between wing chord and flow direction relative to the wing) of 90°, but the cupped shape of the flexible wing results in a slightly lower angle of attack in that wing (approx. 80° at the span position of maximum membrane bulge). Although this suggests some potential difference in lift generation between the wings, lift to drag ratios are very low at high angles of attack indicating an insignificant effect on the results [16]. Furthermore, if lift contributes to the force production, the direction of the resultant force in the flexible wing would change relative to the rigid wing, something we did not find to be the case. The performance boost of a cupped wing clap would further suggest that, in addition to butterflies, other animals that may use clapping (e.g. fish [17] and frogs [18]) could have evolved the use of a cupped wing, fin or foot shape, which would increase the performance of the clap. Our results clearly show that there is a benefit of a cupped clap, such as used by butterflies, but how to optimize it remains a question for future studies. Our membrane wing represents the simplest version of a flexible wing, and understanding the impact of the material properties of the wing (including the effects on other phases of the wingbeat) through affecting both degree of cupping and rebound ability (i.e. ability to close the space between the wings) at the final stages of the clap or even active control of the final stages of a cupped clap will require more advanced robots (e.g. similar to the pectoral fin robot [19]) or computational modelling [20] that can control more aspects of the clapping motion. A more advanced clapper would also allow for studying the effect of two wing pairs interacting rather than using a single wing pair as in our clapper. For example, we note that during the clap the forewings clap together before the hind wings and then start to peel apart, starting the downstroke, but keeping the gap between the forewings closed while the hindwings complete the clap (figure 3). Yet another issue to explore is the effect of wing closeness during the clap, something that, given the correlation with wing amplitude, is best explored comparing single wing performance with clapping pairs of wings [21]. However, the fact that even our extremely fundamental variant of a clapping wing configuration shows such a clear improvement in performance due to flexibility of the wing alone suggests that a more fine-tuned system has the potential to reap even larger benefits and therefore provide selective advantages to animals.

Since Weis-Fogh's iconic paper [3] describing the clap mechanism in insects, it has been generally viewed as an important part of insect aerodynamics, but no quantitative aerodynamic studies of the wing clap have previously been performed on freely flying animals. Our data clearly demonstrate the use of upstroke claps in butterflies. Furthermore, our results show that the butterflies perform the clap in a much more sophisticated way than has ever been known and in our study the upstroke and clap provide all, or nearly all, of the thrust generated during take-off (figure 2*a*). We also show that how the butterflies move their wings correlates with thrust generation (figure 2*c*). Our results demonstrate a separation in function between the downstroke and upstroke, with downstroke providing mainly lift and upstroke and clap producing mainly thrust. The alternating direction of the force vector during downstroke and upstroke is in line with expectations based on the body pitch changes through the wing stroke, where the body is pitching down during the downstroke and pitching up during the upstroke, as reported elsewhere [5,12]. We would suggest that this large angle pitching, that may be unique to butterflies, would increase the cost of flight but also points to the importance for the animals to direct the clap force vector in a useful direction, e.g. [22].

Butterflies have a unique wing shape among nature's flyers. They occupy an extreme place in the morphospace of flying animals, with unusually large wings relative to their body size (very low wing loading) and very short and broad wings [23] (low aspect ratio - span/mean chord; electronic supplementary material, figure S6). However, we have a relatively poor understanding of the mechanistic basis for why butterflies have evolved the extreme relative wing size and shape they have. Large wings with low wing loading, as found in butterflies, allow for slow flight, but low aspect ratio wings have a low span efficiency (efficiency of lift production) and relatively high profile drag (wing drag) resulting in aerodynamically inefficient flight [24,25]. There is some consensus that the large wings of butterflies allow for erratic flight, compared to other insects, and we would suggest that the ability to perform a powerful, thrust generating, clap can be key to this erratic flight and hence escaping a predator in butterflies. Low aspect ratio wings have previously been suggested to improve wing clapping [21] and together with our findings this suggests that the aerodynamic function of wing clapping is crucial to understand the evolution of the extreme position of butterflies in the morphospace of flying animals (electronic supplementary material, figure S6). Also, animals do not only use their wings to clap but also to generate lift. The triangular wing shape used in our clapper is distinctly different from the square wings in previous clapper studies (yet producing an effective clap) and more similar to butterfly wings. The triangular shape gives a higher aspect ratio of the wing than square plates, which would result in more efficient lift production during the downstrokes, providing a wing more suitable for the different phases of the wingbeat. However, there is substantial variation in wing shape among butterflies that partially link to ecology and habitat [24] and further exploration of the effects of this morphological variation on clapping performance, and the link between use of claps and wing shape, is warranted. For example, we would predict that species with higher aspect ratio wings use wing claps less often, since these wings indicate a selection for more efficient lift production instead.

Our results also have implications for design of man-made propulsion systems, as used by unmanned air vehicles [26–28] or underwater drones [29]. Some such devices already use propulsion systems based on a wing clapping motion, e.g. DelFly, NUS-Roboticbird and others [26–28,30]. Although clapping flexible wings in DelFly have been suggested to generate potential benefits [31], the mechanism suggested (improved wake capture when the wings move apart after the clap) is fundamentally different from the mechanism found in this study (improved effectiveness and efficiency of the clap itself). Careful tuning of design and material properties of the wings and fins of flapping drones, to achieve a cupped clap, may thus dramatically improve efficiency and thereby flight/swim duration and range, which are currently among the most challenging problems to solve for these systems.

## Supplementary Material

Supplemental Figures and table

## Supplementary Material

Movie S1

## Supplementary Material

Movie S2

## Supplementary Material

Movie S3

## Data accessibility

The vector data that support the findings of this study are available in Dryad (https://datadryad.org/stash/share/Twr3ItANZYFugcPLcDrNaiKFhA8QSjf2KJr-kBRuwNQ). The Matlab codes used to analyse the data will be available from the authors upon request.
