# Supplementary material for: Butterflies fly using efficient propulsive clap mechanism owing to flexible wings
Source: J R Soc Interface. 2021 Jan 20;18(174):20200854. doi: 10.1098/rsif.2020.0854 (PMC7879755; doi:10.1098/rsif.2020.0854)
Supplement: Supplemental Figures and table [file rsif20200854supp1.pdf]

Supplementary materials:

Butterflies fly using efficient propulsive clap mechanism owing to flexible wings

L. Christoffer Johansson and Per Henningsson

Department of Biology, Lund University, *Ecology Building,*

Sölvegatan 35, 223 62 Lund, Sweden.

Content:

- Supplementary figures and tables
    - Figure S1 - Setup and methods.
    - Figure S2 - Morphology and kinematics.
    - Figure S3 - Clapper design and performance.
    - Figure S4 - Wake structure, 3D rotatable.
    - Figure S5 - The wake vortices of analyzed sequences.
    - Figure S6 - Butterfly morphology in relation to other flying animals.
    - Table S1 - Morphological details.
- References

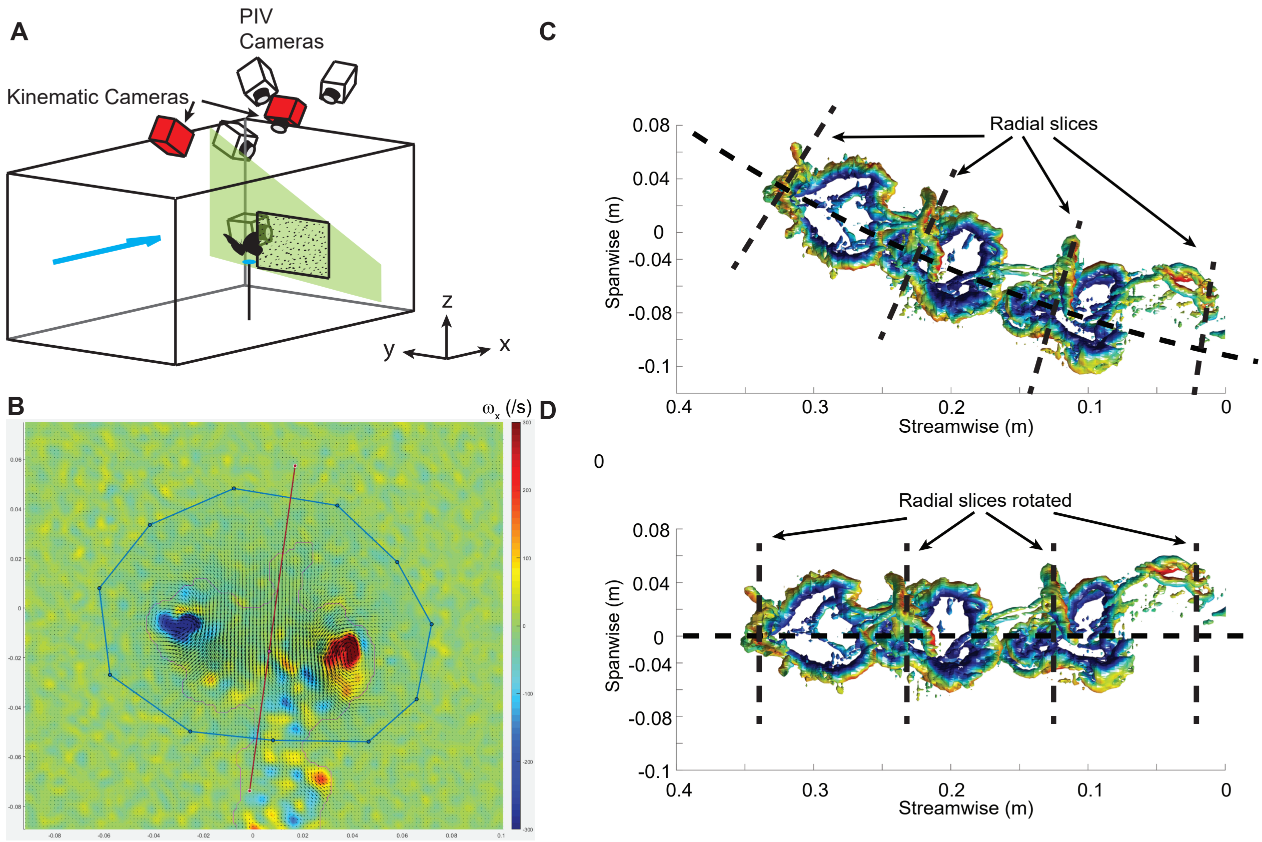

Fig. S1. Setup and methods. Schematic illustration of the experimental setup for tomoPIV and kinematics study of butterflies in the windtunnel (**A**). Two sets of cameras were used, one to film the kinematics (two cameras, red) and one for the tomographical particle image velocimetry measurements (four cameras, white). The PIV cameras filmed particles suspended in the air and illuminated by the laser (green). Flow direction of the tunnel is indicated by the blue vector. The vector fields were analyzed using a combination of manual and automatic masking of the wake structures (**B**). The automatic mask is seen as a red line around the wake vortices and the manual mask as a blue polygon. The red line was used to measure the tilt of the wake in relation to the horizon. The image shows a sample from a downsroke wake with blue background indicating clockwise vorticity (angular rotation of the flow) and red counter clockwise vorticity in the straitened coordinate system (x’,y’,z). Straightening of the wake for analysis (**C** and **D**). Top-down views of vorticity iso-surface plots colored by downwash of an example sequence. Panel (**C**) show before and panel (**D**) after ‘straightening’ of the wake for analysis purposes. The dashed arc in the upper panel shows how the fitted circle approximates the flight path from the original wake and the four radial dashed lines illustrates how slices where cut through the volume. In reality each sequence was sliced with the same number of slices as the original number of frames.The dashed horizontal line in the bottom panel illustrates how the same arc, now a straight line, cuts through the wake after it has been straightened out by rotation of the individual PIV volume slices along the sequence.

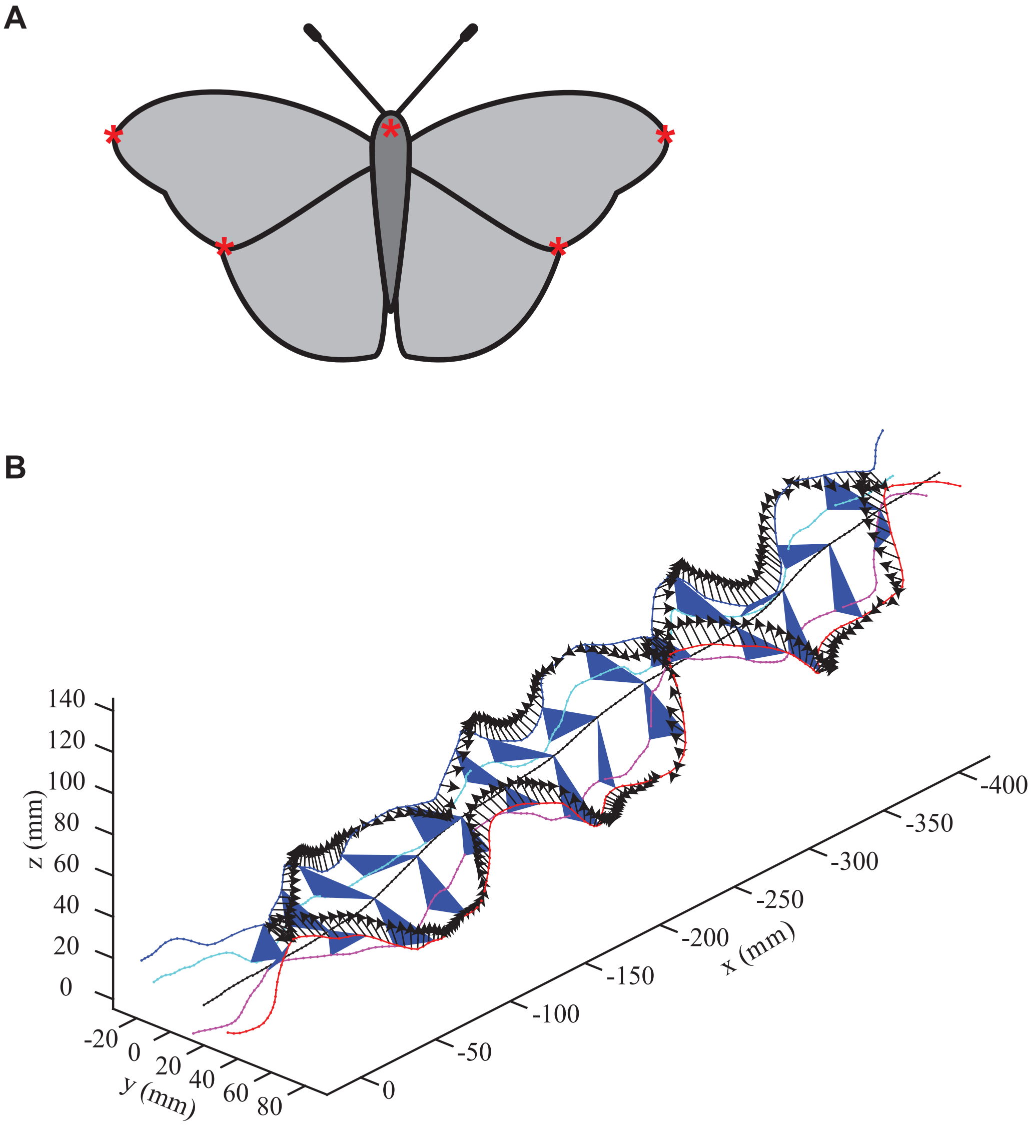

Fig. S2. Morphology and kinematics. Illustration showing the five digitized points on the butterflies in the high-speed movies used for the kinematic analysis (**A**). One point on top of the head, one on each tip of the forewings and one on each tornus of the forewings. The 3D reconstructed motion of the digitized points are shown as lines (**B**), head (black), left wing tip (blue), left tornus (cyan), right wing tip (red) and right tornus (magenta). Every tenth frame each front wing is represented by a blue triangle. The normal to this triangle is illustrated by black vectors, showing the orientation of the front wings at each frame.

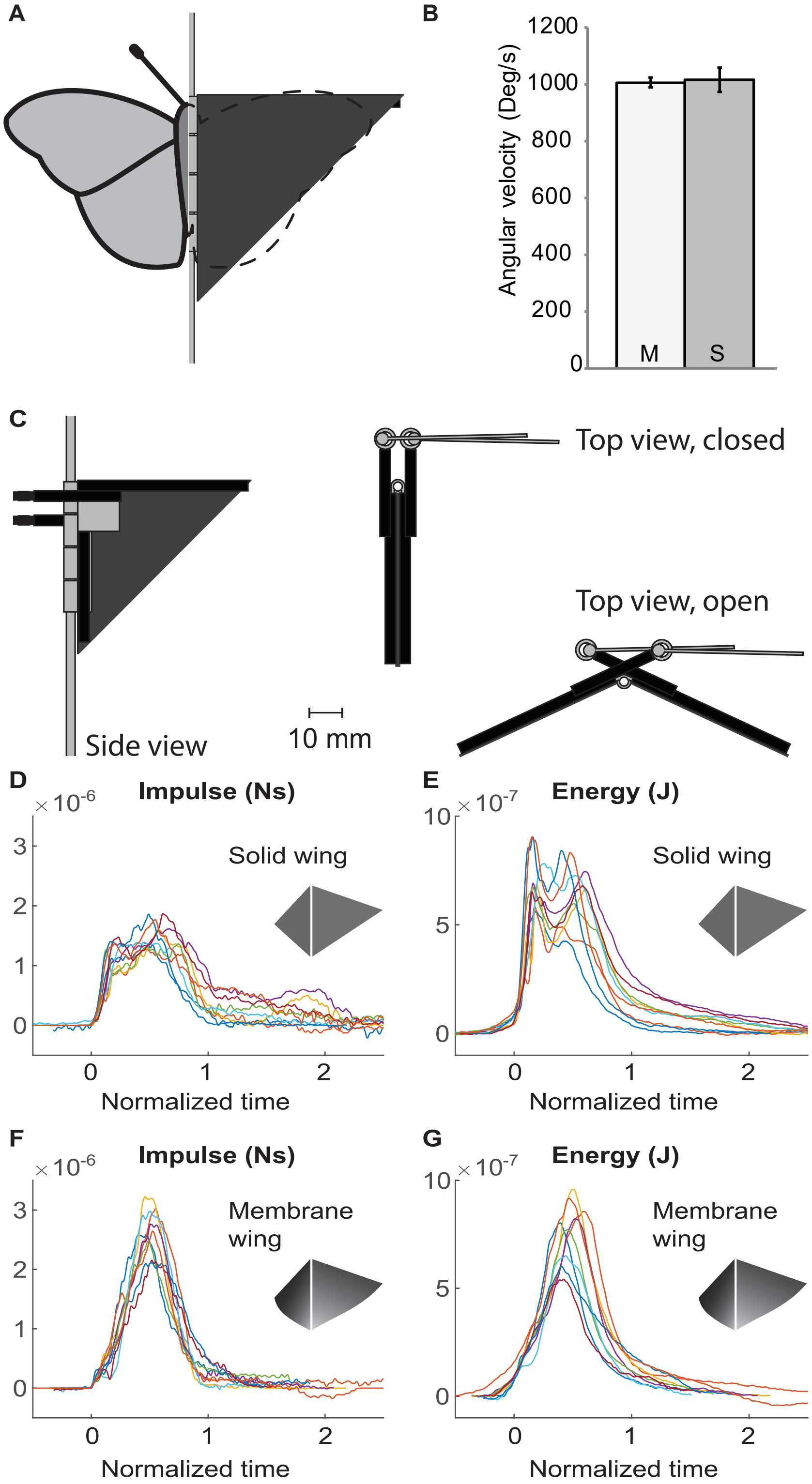

Fig. S3. Clapper design and performance. (**A**) Outline of butterfly and the triangular clapper wing. (**B**) Clapper wing angular velocity. Mean angular velocity of the membrane (M) and solid (S) clapper wings during the measurements. Error bars show standard error of mean. The similarity between the averages and the small standard error show that the performance of the clapper was consistent. (**C**) Drawing of our clapper with membrane wing. Side view and top view with closed and open wings. Black rods are made from 3 mm carbon fiber rods and grey parts are made of steel. The membrane is a 0.25 mm thick latex sheet and is supported along the vertical axis and the leading edge (top), while the trailing edge of the wing is free to deform. (**D-G**) Flexible wing improve impulse and efficiency of wing clap. How the impulse varies over a normalized clap wake differs between a solid wing (**D**) and a flexible membraneous wing (**E**) with indications of a double peak in the solid wing curve. The associated energy in the wake (**F**, **G**) show a similar pattern as the impulse. The x-axis represents normalized time, where 0 is the time of first impulse in the wake and 1 is the time the distinct clap wake has passed through the measurement plane. The colored lines represent the individual measurements for each type of wing (N=9 for each wing).

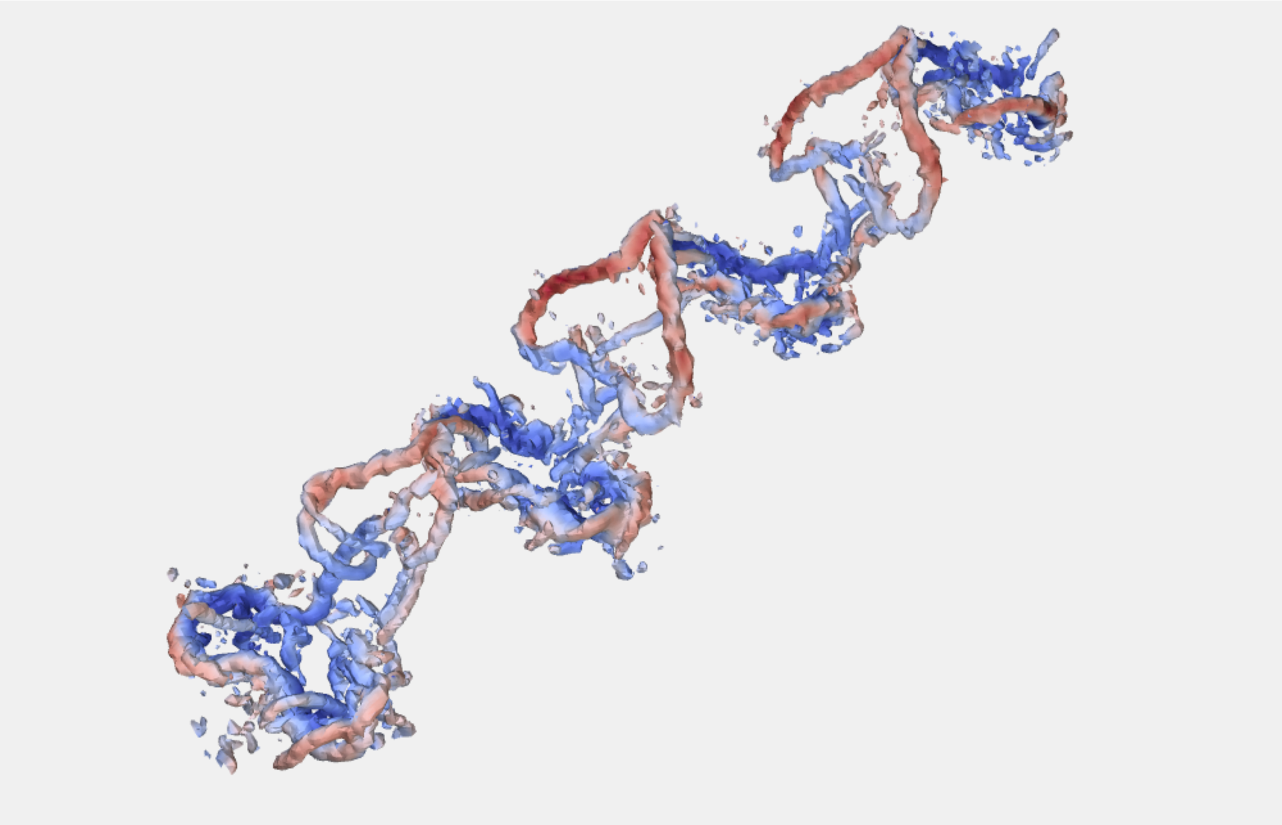

Fig. S4. Wake structure, 3D rotatable. The wakes of the downstrokes are more or less horizontally oriented indicating generation of mainly vertical force. The upstroke wakes are instead vertically oriented, indicating mainly thrust production. Wake vortices are illustrated as isosurfaces of Q, colored by downwash and represents the same wake as in Fig. 1 (see Fig. 1 for colorbar). The wake in the start image is seen obliquely from above and behind, with the butterly flying into the image and to the right. Rotate the image to see other views.

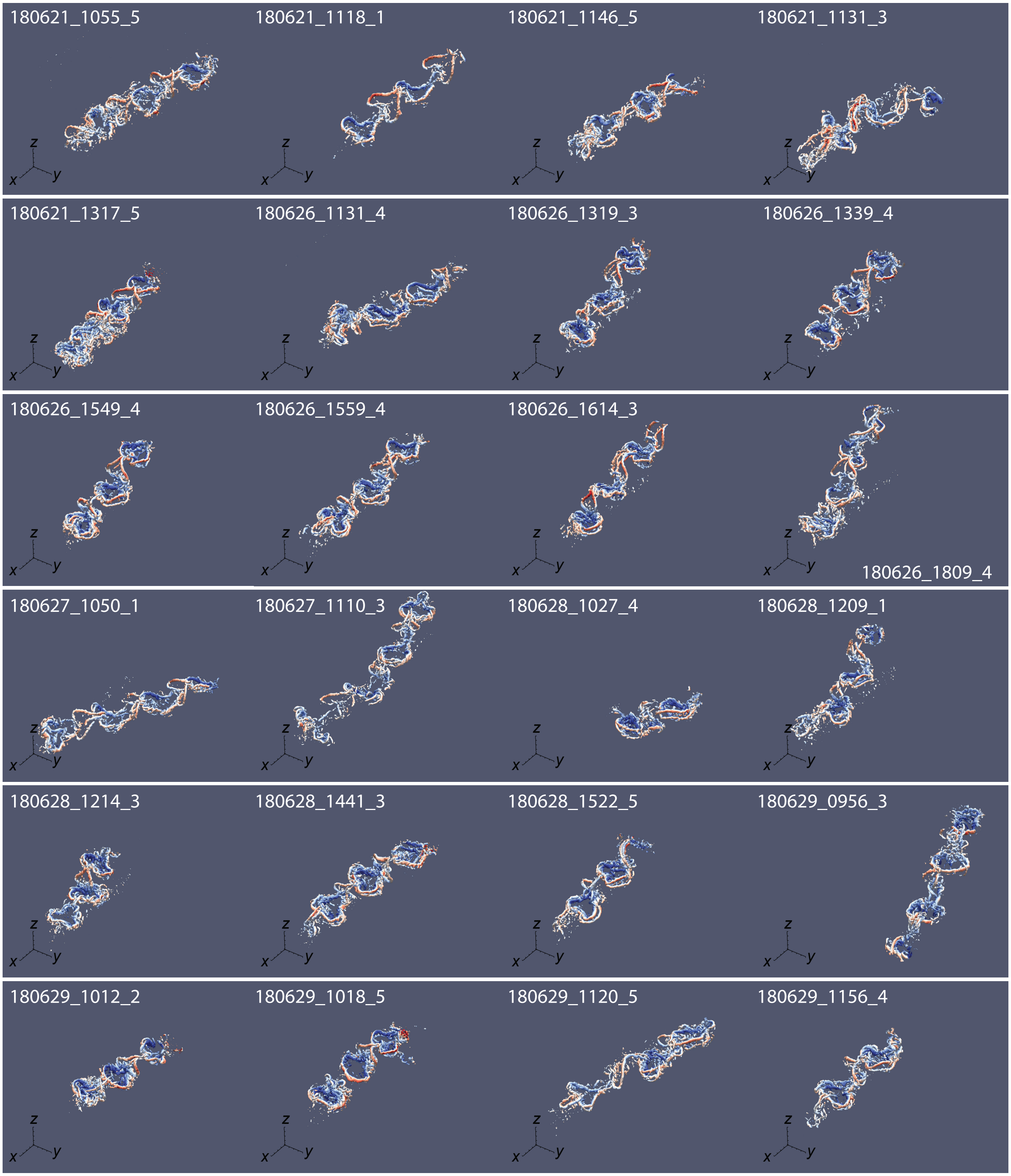

Fig. S5. The wake vortices of all analyzed sequences except the one in Fig. 1. Wake vortices are illustrated as iso-surfaces of Q (=4000), a measure of rate of rotation relative to rate of shear in the flow, colored by downwash (w). The wake is seen obliquely from above and behind, with the butterfly flying into the image and to the right, performing various degrees of turning. The distance between each axis tick in the coordinate system is 0.0125 m.

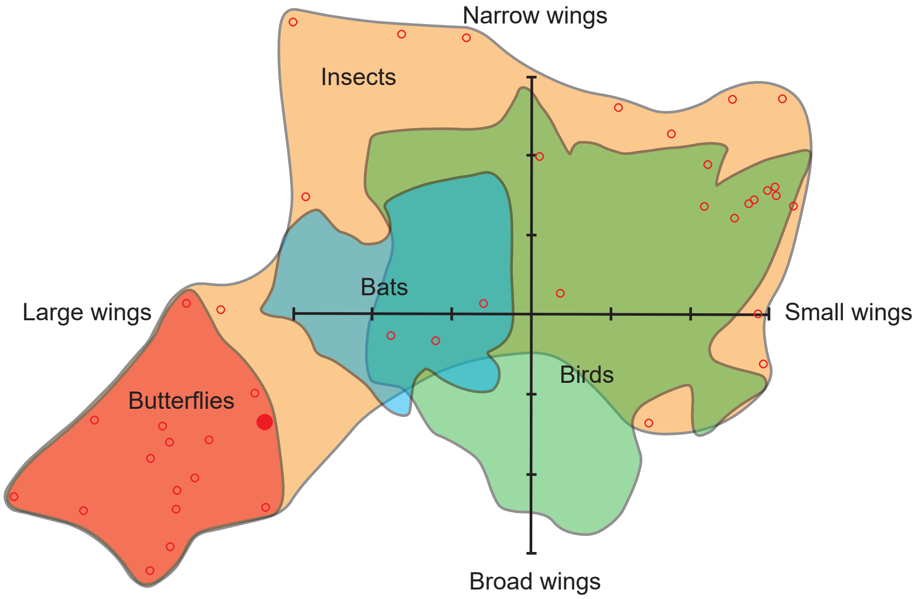

Fig. S6. Butterfly morphology in relation to other flying animals. Butterflies occupy an extreme part of the morphospace not represented by any other group of flying animals. Wing shape (PC3 = $\ln(4.39\cdot10^{-4}\cdot m^{-0.17}\cdot b^{-6.84}\cdot S^{-3.55})$,where *m* is body mass, *b* wingspan and *S* wing area) and relative size of wings (PC2 = $\ln(8.35\cdot10^{-2}\cdot m^{2}\cdot b^{-2.28}\cdot S^{-1.53})$) are represented by PCA scores from Rayner (1). Bird morphospace is redrawn from Rayner (1) and bat morphospace from Norberg and Rayner ((2), as presented by (1)). Insect datapoints (red circles) are calculated based on limited morphometric data set from (3-7). The species in this study, *Argynnis paphia*, is represented by a filled circle.

Table S1.

Morphological details of the six individuals of silver-washed fritillaries used in the experiments.

Ind	Sex ♂/♀	Mass (mg)	Weight (mN)	Span (mm)	Area (mm^2)	Aspect ratio	Wing loading (kg/m^2)	Wing loading (N/m^2)
1	♂	185.5	1.82	67	1353	3.3	0.137	1.34
2	♂	210.5	2.06	72	1614	3.2	0.130	1.28
3	♀	198.9	1.95	67	1310	3.4	0.152	1.49
4	♀	227.3	2.23	72	1584	3.3	0.143	1.41
5	♀	187.1	1.83	66	1320	3.3	0.142	1.39
6	♂	208.6	2.05	68	1344	3.4	0.155	1.52

References

1 Rayner, J. M. V. in *Curr. Ornithol.* Vol. 5 (ed R. F. Johnston) 1-66 (Plenum Press, 1988).

2 Norberg, U. M. & Rayner, J. M. V. Ecological morphology and flight in bats (Mammalia; Chiroptera): Wing adaptations, flight performance, foraging strategy and echolocation. *Phil. Trans. R. Soc. Lond. B* **316**, 335-427 (1987).

3 Ellington, C. P. The aerodynamics of hovering insect flight. II. Morphological parameters. *Phil. Trans. R. Soc. Lond. B* **305**, 17-40 (1984).

4 Dudley, R. Biomechanics of flight in neotropical butterflies: morphometrics and kinematics. *J. Exp. Biol.* **150**, 37-53 (1990).

5 Ahmad, A. Comparative study on flight surface and aerodynamic parameters of insects, birds and bats. *IJEB* (1984).

6 Yager, D. D. & May, M. L. Ultrasound-triggered, flight-gated evasive maneuvers in the praying mantis Parasphendale agrionina. II. Tethered flight. *J. Exp. Biol.* **152**, 41-58 (1990).

7 Johansson, L. C. *et al.* Elytra boost lift, but reduce aerodynamic efficiency in flying beetles. *J. R. Soc. Interface* **9**, 2745-2748 (2012).
